# *De Novo* Sequencing of *Astyanax mexicanus* Surface Fish and Pachón Cavefish Transcriptomes Reveals Enrichment of Mutations in Cavefish Putative Eye Genes

**DOI:** 10.1371/journal.pone.0053553

**Published:** 2013-01-09

**Authors:** Hélène Hinaux, Julie Poulain, Corinne Da Silva, Céline Noirot, William R. Jeffery, Didier Casane, Sylvie Rétaux

**Affiliations:** 1 DECA Group, N&D Laboratory, CNRS, Gif sur Yvette, France; 2 Génoscope-CEA Sequencing Center, Evry, France; 3 INRA Bioinformatics Platform, Toulouse, France; 4 Department of Biology, University of Maryland, College Park, Maryland, United States of America; 5 LEGS, CNRS Gif sur Yvette and Université Paris Diderot, Sorbonne Paris Cité, France; The Scripps Research Institute, United States of America

## Abstract

*Astyanax mexicanus*, a teleost species with surface dwelling (surface fish) and cave adapted (cavefish) morphs, is an important model system in evolutionary developmental biology (evodevo). *Astyanax* cavefish differ from surface fish in numerous traits, including the enhancement of non-visual sensory systems, and the loss of eyes and pigmentation. The genetic bases for these differences are not fully understood as genomic and transcriptomic data are lacking. We here present *de novo* transcriptome sequencing of embryonic and larval stages of a surface fish population and a cavefish population originating from the Pachón cave using the Sanger method. This effort represents the first large scale sequence and clone resource for the *Astyanax* research community. The analysis of these sequences show low levels of polymorphism in cavefish compared to surface fish, confirming previous studies on a small number of genes. A high proportion of the genes mutated in cavefish are known to be expressed in the zebrafish visual system. Such a high number of mutations in cavefish putative eye genes may be explained by relaxed selection for vision during the evolution in the absence of light. Based on these sequence differences, we provide a list of 11 genes that are potential candidates for having a role in cavefish visual system degeneration.

## Introduction

*Astyanax mexicanus* is a characiform fish consisting of distinct surface dwelling (surface fish) and cave adapted (cavefish) forms. The ancestors of cavefish were isolated in caves about a million-year ago (Ma) and have since adapted to this extreme environment, which is characterized by constant darkness and food scarcity. Cavefish differ from their surface counterparts in numerous morphological, physiological and behavioral traits, the most striking being that cavefish lack functional eyes and are de-pigmented, and generally have lower metabolic rates than surface fish [1]–[6]. Twenty-nine different cavefish populations have been discovered so far, and some of them were derived independently, allowing the study of parallel evolution [7]. Cavefish and surface fish are inter-fertile, making *Astyanax mexicanus* an outstanding genetic model for microevolution studies [1]. All the phenotypic changes in cavefish, including the loss of eyes and pigmentation, may be explained by different evolutionary mechanisms. The two main hypotheses are: (1) positive selection, either direct or indirect, for traits that are beneficial in the dark, (2) neutral evolution by genetic drift, for traits that are not under selection [8], [9].

Neither genomic nor transcriptomic data are currently available for *Astyanax mexicanus.* The closest model species with a sequenced genome is the zebrafish *Danio rerio*, a cypriniform. The common ancestor of characiforms and cypriniforms diverged at least 100 Ma [10], [11] and could even be more distantly related (>200 Ma), rendering some comparisons difficult [12]. The genetic bases of adaptation to life in caves have thus remained elusive. From studies in other model organisms, it was proposed that phenotypic evolution can be explained in part by changes in non-coding regulatory sequences: for example, in stickleback *Gasterosteus aculeatus*, pelvic spine reduction during the transition from marine to freshwater environments is due to the deletion of a *Pitx1* enhancer [13], [14]. However, phenotypic changes can also be based on mutations in coding sequences. For instance, the reduction or loss of pigmentation in *Astyanax mexicanus* cavefish is due to mutations in the *Mc1r* and *Oca2* coding sequences [15], [16]. A few other coding sequences were investigated in *Astyanax* in attempts to understand the genetic bases for cavefish eye degeneration. On the one hand, the “master gene” for eye development, *Pax6*, was found to be identical in the two populations [17], while on the other hand, opsin gene sequences were found to accumulate C->T transitions in cavefish, as a signature of pseudogenes formation [18]. These case studies are still limited to a small number of genes, due to the lack of sequence data. This situation will change in a near future, due to the ongoing Pachón cavefish genome project at the Washington University in Saint Louis.

In the context of a paucity of sequence information, understanding the evolutionary history of *Astyanax mexicanus* populations is also challenging. Relying on 6 microsatellite loci and mitochondrial DNA, it was shown that not all cave populations share the same origin [19]. More recently, using 26 microsatellite markers, Bradic et al. proposed a model with five independent origins of cave-adapted *Astyanax* in Mexico, with two invasion “waves” of surface fish into the subterranean environment establishing “old” and “new” cave populations [7]. Pachón cavefish, which shows the most severe eye degeneration and de-pigmentation phenotypes is the most studied cave population and belongs to the “old” populations [7]. In previous studies of the various cavefish populations, the genetic diversity was generally found to be lower in cavefish than in surface fish [7], [19], possibly resulting from small effective population sizes because of food and space limitations or from population bottlenecks due to sporadic environmental degradations [20]. Obtaining large sequence datasets on *Astyanax mexicanus* surface fish and cave populations to assess their genetic diversity would therefore also help understand their evolutionary history.

Here we have sequenced cDNA libraries from several different developmental stages of *Astyanax mexicanus* surface fish and Pachón cavefish. The crucial need for long transcript sequences and the lack of a close reference genome led us to use the Sanger sequencing method. The inclusion of different developmental stages allowed scanning most of the developmental transcriptome, as well as the successive steps of eye development and degeneration in Pachón cavefish. About 200,000 clones were sequenced, providing a new resource for the *Astyanax* research community. These transcriptomic sequences were then used to compare the level of polymorphism in the coding sequences of the two *Astyanax* morphs at a larger scale than what was previously possible [7], and to identify fixed differences in coding sequences between surface fish and cavefish, which are candidates for being involved in some of their phenotypic differences.

## Methods

### Ethics Statement

Animals were treated according to the French and European regulations for handling of animals in research. SR’s authorization for use of animals in research is number 91–116. Laboratory study uses exclusively embryos and early larvae from aquatic vertebrate (non-mammalian) animals and therefore did not require special authorizations. Field sampling was conducted with Mexican Permit Number 040396-213-03 granted to W. R. Jeffery. Fish were caught using nets. A small (4 mm2) tissue sample was excised from the caudal fin and stored in 100% ethanol before release of the fish at the point of capture. All efforts were made to minimize suffering.

### Biological Material

*Astyanax mexicanus* surface and Pachón cavefish were obtained from the Jeffery lab (University of Maryland, College Park, MD) in 2004. Surface fish had initially been collected in San Solomon Spring, Balmorhea State Park, Texas. In our Gif sur Yvette facility, fish are maintained and bred at 23°C (cavefish) or 26°C (surface fish) on a 12∶12 hour light/dark cycle in tap water. They are kept in groups of ∼30 fish in large 120–200 liters tanks. Spawning is induced in these breeding groups by changing tank water and shifting temperature (−4°C for surface fish, +4°C for cavefish). No selection for some reproductive individuals is performed, and breeding individuals are mixed, maximizing the retention of genetic diversity from generation to generation. We estimate that a maximum of 5 laboratory generations have occurred since the initial capture of surface fish and cavefish in the wild in 2000.

### cDNA Libraries

Fish embryos and larvae were anaesthetized with MS222 (Sigma), immediately immersed in Trizol (Invitrogen), and frozen at −80°C. Fifty to 200 embryos/larvae originating from several independent spawns were pooled for each developmental stage of the two morphs. RNA extraction was performed using Trizol following the manufacturer’s instructions.

Eight libraries were constructed in a pCMV-SPORT6 derivative (polylinker region modified to include SfiI-sites for compatibility with directional cloning). This vector includes a CMV promoter for expression and a T7 promoter for antisense probe production. RNA was reverse-transcribed with Mint reverse transcriptase (MMLV-based, Evrogen) and cDNA was ligated into pCMV-Sport6 vector by LGC Genomics (Berlin). The Mint Universal cDNA Synthesis Kit and the Trimmer Normalization Kit (both from Evrogen) were used. The 8 ligation products corresponding to the 8 libraries (2 normalized, 6 non-normalized) were transformed into E. Coli DH10B phageT1 resistant bacteria at the Genoscope (Evry, France). Clones were arrayed onto 384 multiwell plates and sequenced using Sanger technology.

### Cleaning of the ESTs Sequences

198,380 Sanger ESTs (Expressed Sequence Tags) were obtained from the sequencing of the 8 libraries. The mean length of reads was 1,364 bp. 44 additional *A. mexicanus* mRNAs were also recovered from GenBank database (Table S1A).

Sanger sequences were cleaned with Seqclean with the following options: i\ vector sequence pCMV_sport6, and ii\ contaminant sequences of yeast, E. coli536, and phage sequences from Genbank phage division. Then, low quality sequences at the extremities and very short sequences were removed with Prinseq [21], with the following parameters: window of 30, step of 5, minimal length 100 bp. A description of the libraries generated before and after cleaning is provided in Fig S1. ESTs sequences were submitted to Genbank under accession numbers FO203528 to FO393391.

### Assembly and Annotation Procedure

Assembly of Sanger sequences was carried out using TGICL software [22]. This software uses the CAP3 assembler [23] that takes into account the quality of sequenced nucleotides into the computation of the alignment score. The choice for using TGICL software is justified by its good performance for *de novo* assembly of long ESTs [24]. Moreover, TGICL generates less chimeric contigs than the more recent Newbler software [25]. 44,145 contigs were generated, including 29,114 singlets.

These contigs were annotated with the Biotoul platform pipeline, firstly performing BLAST against the following databases: i\ Reference databases: UniProtKB, RefSeq Protein and RNA, Pfam; ii\ TIGR fishes databases; iii\UniGene fishes species; iv\ Ensembl fishes Transcripts (a detailed list of databases and versions is given in Table S1B). Gene ontology (GO) terms associated to each contig best hit (from TrEMBL, RefSeq or Swissprot databases) were analyzed using Blast2GO v.2.5.1 [26] to build a pie chart of their frequency distribution.

The repeat sequences were detected by RepeatMasker [27]. All those annotations were loaded into an EnsEMBL-like browser called Contigbrowser. Browsing Unigene *Astyanax* contigs and data mining by BioMart are available at http://genotoul-contigbrowser.toulouse.inra.fr:9099.

### Polymorphism Analysis

Non-singlets contigs were blasted against the zebrafish proteome (Zv9 assembly, downloaded from EnsEMBL [28]), the closest species with a sequenced genome, to annotate them. Only one contig per zebrafish hit was kept, to avoid artificial increase of polymorphism by counting twice the same polymorphic site detected in two different contigs. When multiple contigs blasted on the same zebrafish protein, the contig with the largest depth was selected. In total 6,431 contigs were retained. ESTs for these contigs were modified so that each nucleotide with a quality score below 20 was replaced by ‘N’ and they were then realigned to their respective contig by Megablast (parameters: opening gap cost 0, penalty for mismatch −2), which is a more stringent alignment algorithm than the assembling software. This allowed regions where ESTs did not align properly to the contig to be discarded. Then in the aligned regions, ESTs were compared to the reference contig sequence (BLAST results were processed using the Bio::SearchIO module of BioPerl toolkit [29]). Polymorphism analysis was performed independently for cavefish and surface fish, and the minimal depth for each morph was set to be 4. With a depth of 4, two representatives of the minor allele had to be present at a given position to be considered polymorphic, and the minimal occurrence required for the minor allele increased with the depth D. Two conditions had to be fulfilled for the minor allele not to be discarded: firstly there had to be enough occurrences of this allele to eliminate the likelihood of an error; secondly it had to be present at a frequency that made sense considering the biological samples that were sequenced.

The first threshold was determined by estimating that the rate of error was mostly dependent on the Mint reverse transcriptase, which is supposed to make an error every 30,000 nucleotides [30] (Sanger sequencing error rate is comparatively much lower). To be stringent we estimated that the global error rate was 10^−4^ and used a binomial law to calculate the probability to have k errors at a position of depth D (k being the occurrence of the minor allele). To take into account the fact that in each contig errors can occur at all sites, we used the probability calculated above and a binomial law to calculate the probability to have at least one position with k errors among all the positions of the contig. As the mean length of contigs is 985 bp, we calculated this probability for a length of 1,000 bp. If the occurrences of the minor allele could be explained by errors with a probability higher than 0.01, the putative polymorphic site was not retained.

Regarding the second threshold, we estimated that at least 10 individuals of each morph had been involved in the breeding that gave rise to the sampled embryos. With a stringent assumption (no more than 10 individuals), 20 alleles at most would be present in the sampled embryos. It would thus be impossible to observe an allele with a frequency lower than 0.05.

The polymorphisms were then sorted into different classes: (1) “shared polymorphism” for positions at which both cavefish and surface fish sequences were polymorphic, and with the same alleles, (2) “divergent polymorphism” for positions where both cavefish and surface fish sequences were polymorphic but with different alleles, (3) “polymorphism in one morph only” when either cavefish or surface fish was polymorphic and the depth was equal or higher than 4 in the other morph, (4) “polymorphism in one morph, unknown status for the other morph” at positions where the apparently non-polymorphic morph had insufficient depth.

Fixed differences between the two morphs were also analyzed at positions where the depth for each one was at least 4, and where all cavefish shared the same allele and all surface fish shared another allele. Cavefish and surface fish transcripts were then translated into proteins and aligned with the corresponding zebrafish protein, which allows for eliminating the contig regions that were non-coding. The coding regions of the translated surface fish and cavefish contigs were then compared in order to identify non-synonymous substitutions. The amino-acid substitutions between the two morphs were oriented using *Danio rerio* proteins as outgroups. In order to detect radical substitutions, amino acids were categorized into 6 distinct classes: hydrophobic, aromatic, polar neutral, acidic, basic, and proline. The expression pattern of the genes with radical mutations was searched for using the Zfin database [31]. The enrichment for genes expressed in the eyes was statistically tested using Fisher’s exact test. For genes with mutations which had occurred in the cavefish lineage, the presence of conserved domains was identified using the Prosite [32] and NCBI Conserved Domain databases [33]. Several files containing a description of detected polymorphisms can be downloaded from the *Astyanax* browser.

The same approach was applied to detect population-specific indels, but no such indels were found in the contig coding sequences aligned to zebrafish proteins.

Orthology relationships were verified for all the cited potential candidate genes using Neighbor Joining phylogenetic analysis with Mega5 [34] (not shown).

Non-singlet contigs used in the polymorphism analysis were annotated for gene ontology term (GO term) using EnsEMBL BioMart, and contigs with substitutions modifying the protein sequence were analyzed for GO term enrichment using conditional hypergeometrical test of GOstats R package [35].

### Polymerase Chain Reaction on Genomic DNA

*Astyanax mexicanus* fin clips were collected in the wild in March 2008 by Bill Jeffery and Yoshiyuki Yamamoto (Mexican Permit Number 040396-213-03 granted to W. R. Jeffery). Fish were caught using nets. A small (4 mm2) tissue sample was excised from the caudal fin and stored in 100% ethanol before release of the fish at the point of capture. Surface fish fin clips originate from the Rio Valles near the village of Micos (San Luis Potosi, Mexico). Genomic DNA was extracted from these fin clips with standard phenol-chloroform protocol.

Srd5a PCR was performed using a first set of primers allowing the amplification of exons 4 and 5 (Fw 5′ GGGTGTTTGTCACTCTTCTGC 3′ and Rv 5′ GCCCTCAGTACCTCAGTGCA 3′) and then a semi-nested PCR using a different Fw primer (5′ CGTGACTACGCTGGTTGGGC 3′). PCR fragments were sequenced using the following primer: 5′ GGTCCGGTTTGTTCCTGTCTGC 3′ by GATC Company.

## Results and Discussion

### Eight *Astyanax* Cavefish and Surface Fish cDNA Libraries

Our aim was double: (1) to generate cDNA libraries from biologically relevant developmental stages for surface fish and cavefish as a clone resource, and (2) to generate transcriptome data for analysis of the genetic basis of cavefish evolution. Therefore, we extracted total RNA from 4 different stages of surface fish and Pachón cavefish embryos and larvae chosen according to the *Astyanax mexicanus* developmental staging table [36]: (1) gastrulae to neurulae (6–16 hpf), (2) hatched larvae (24–36 hpf), (3) swimming larvae (48–60 hpf) and (4) juveniles (2 weeks old). The first stage corresponds to the period in which the optic cup and lens placode are formed in surface fish and cavefish embryos. During the second stage, the cavefish but not the surface fish lens initiates apoptosis and arrests in differentiation. During the third stage, the retina begins to degenerate in cavefish, while in surface fish it is becoming functional. During the fourth stage, the cavefish eye continues to degenerate, whereas the surface fish eye undergoes normal growth. Thus the different stages chosen correspond not only to major developmental events but also to important time points in cavefish eye degeneration.

Fifty to 200 embryos/larvae originating from several independent spawns were pooled for each developmental stage and each morph, to be certain that the libraries were representative of the genetic diversity in the two *Astyanax* morphs. Indeed, in our fish facility, breeding occurs in large groups of approximately 30 individuals, so that every spawn is likely to contain the offspring of multiple matings and thus retains the extent of the genetic diversity of their wild-caught ancestors in the RNA samples used to prepare the libraries.

**Figure 1:**
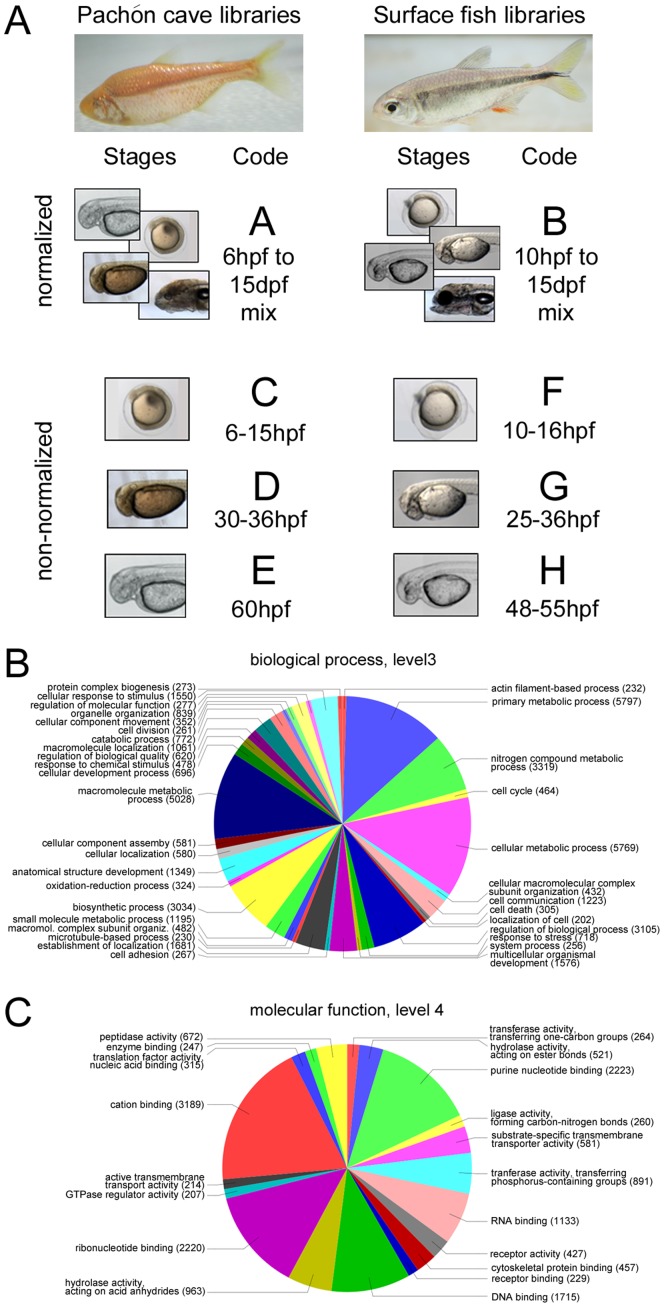
Composition and quality of the Astyanax cDNA libraries. A: Composition of the 8 Astyanax developmental cDNA libraries. Biological process (B) and molecular function (C) gene ontology pie charts of the 17,152 contigs annotated for GO term.

Eight cDNA libraries were generated, 6 non-normalized and 2 normalized (Fig. 1A). Insertion of the cDNA into the vector was oriented, allowing the expression under control of the CMV promoter for those transcripts that are full-length. The mean insert size of the libraries is 900 bp.

### Assembly and Annotation of the Sanger Sequences

Approximately 19,000 clones of each non-normalized library, as well as 43,000 clones of each normalized library were sequenced by the Sanger method (Fig S1B), and none of these libraries showed saturation (Fig S2), meaning that during the sequencing project when new clones were sequenced, they mostly corresponded to genes that had not been sequenced earlier in the project.

After removal of the vector, polyA sequences and poorly sequenced regions, the resulting ESTs had a mean length of 624 bp. 189,933 ESTs from all libraries were used to build 44,145 contigs. The mean length of the contigs is 985 bp, and the mean depth is 6.8. The contigs were annotated by BLAST analysis against several databases (see Methods and Table S1B). As a result, more than 90% of the contigs were annotated. When blasted against the zebrafish proteome, they corresponded to parts of 11,197 different proteins (among the 41,693 EnsEMBL zebrafish proteins). These proteins are encoded by 10,058 different genes (among the 32,469 EnsEMBL zebrafish genes). If the numbers of genes and proteins are similar in zebrafish and *Astyanax*, these contigs would then represent more than a quarter of the *Astyanax* proteome and a third of its genes (Table S2).

Moreover, 17,152 contigs were annotated for gene ontologies, and these gene ontologies are varied, thus the contigs appear to be representative of the *Astyanax* embryonic/larval transcriptome (Fig. 1BC).

The *Astyanax* transcriptomic sequences are available through the web browser http://genotoul-contigbrowser.toulouse.inra.fr:9099. They were also submitted to Genbank under accession numbers FO203528 to FO393391. They represent the first large scale sequence resource for this model species in evodevo, and will be very useful to perform phylogenetic, expression and function studies.

### Polymorphism Analysis

**Figure 2:**
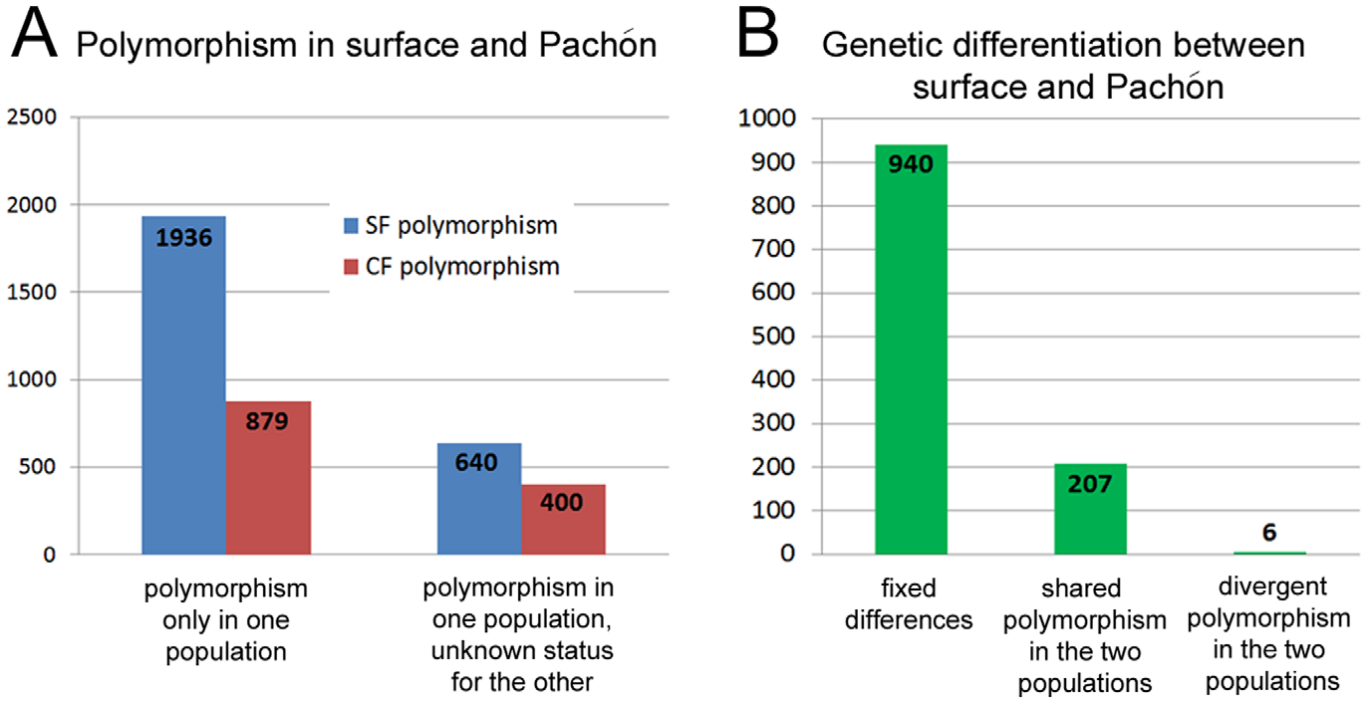
Types of polymorphism uncovered in the surface fish and cavefish transcriptomes. A: Number of polymorphic positions in the nucleotidic sequences of the two Astyanax morphs. B: Number of fixed nucleotide differences, shared polymorphisms and divergent polymorphisms.

To further exploit the *Astyanax* sequence resource, we performed polymorphism analysis (Fig. 2). We blasted the 15,031 non-singlets contigs against the zebrafish proteome and selected one contig per zebrafish hit. Accordingly, 6,431contigs were subsequently analyzed (see Methods).

As expected, polymorphic contigs were built with a relatively high number of ESTs from non-normalized libraries: 65.8% of the ESTs belonging to polymorphic contigs were derived from non-normalized libraries, whereas non-normalized libraries provided only 54.6% of the total number of sequenced ESTs.

Polymorphism was found to be approximately twice as high in surface fish compared to cavefish (Fig. 2A). This result confirms and extends with a large dataset the findings of previous studies based on a few microsatellites [7]. One explanation for this now established tendency is that cavefish have a smaller effective population size than surface fish, and possibly underwent population bottlenecks due to environmental variations. Cavefish and surface fish do share some polymorphic sites, but they have also fixed 940 different alleles (Fig. 2B).

Among the 940 fixed differences, 716 are synonymous. As the closest species (zebrafish) that can be used as an outgroup diverged at least 100 Ma, we assumed that several parallel nucleotide substitutions and reversions may have occurred in each lineage. We thus did not try to infer the direction of these nucleotide changes and did not investigate further the synonymous differences.

**Figure 3:**
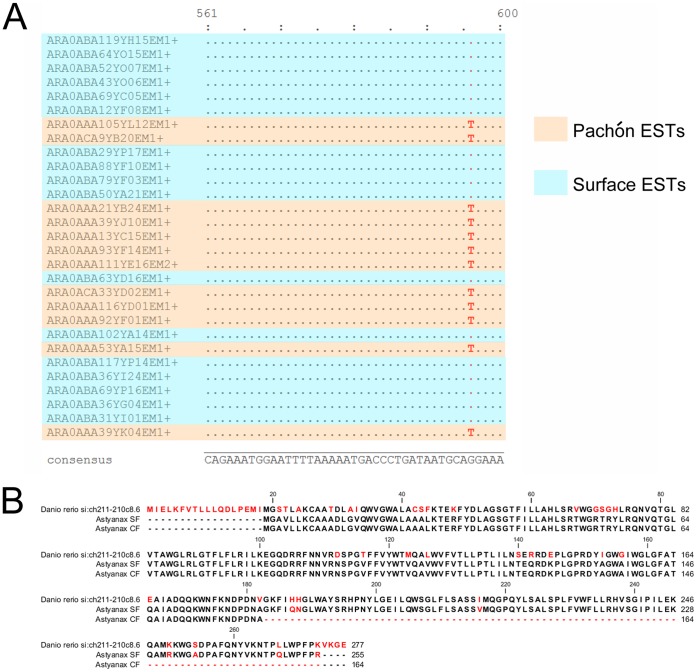
Premature stop codon in a cavefish sequence. A: Alignment of surface fish and Pachón cavefish nucleotide sequences of the si:ch211–210c8.6 transcript. B: Alignment of surface, Pachón and zebrafish translated protein sequences.

However, some of the 224 non-synonymous changes might be responsible for phenotypic differences observed between the two morphs. In addition, a premature stop codon in a cavefish sequence was detected (Fig. 3). The affected gene, a homolog to zebrafish si:ch211–210c8.6, is a member of the srd5a gene family according to our phylogenetic analysis (not shown), which encode enzymes involved in steroid hormones metabolism. We verified that this difference was also fixed in natural populations. We thus amplified exons 4 and 5 of this gene from genomic DNA extracted from fin clips of 4 wild-caught individuals of each morph: this confirmed that the premature stop codon is fixed in the natural Pachón population (not shown).

We also looked for indels specific for one of the two morphs but this analysis did not reveal any indels in coding sequences.

For the 224 amino acid substitutions found, the protein sequences were aligned to zebrafish to infer the direction of the substitutions, based on the principle of parsimony. Among them, 87 mutations had occurred in the cavefish lineage, and 65 mutations had occurred in the surface fish lineage; 72 others could not be oriented because the zebrafish amino acid at the mismatch position was different from both surface fish and cavefish amino acids.

**Figure 4:**
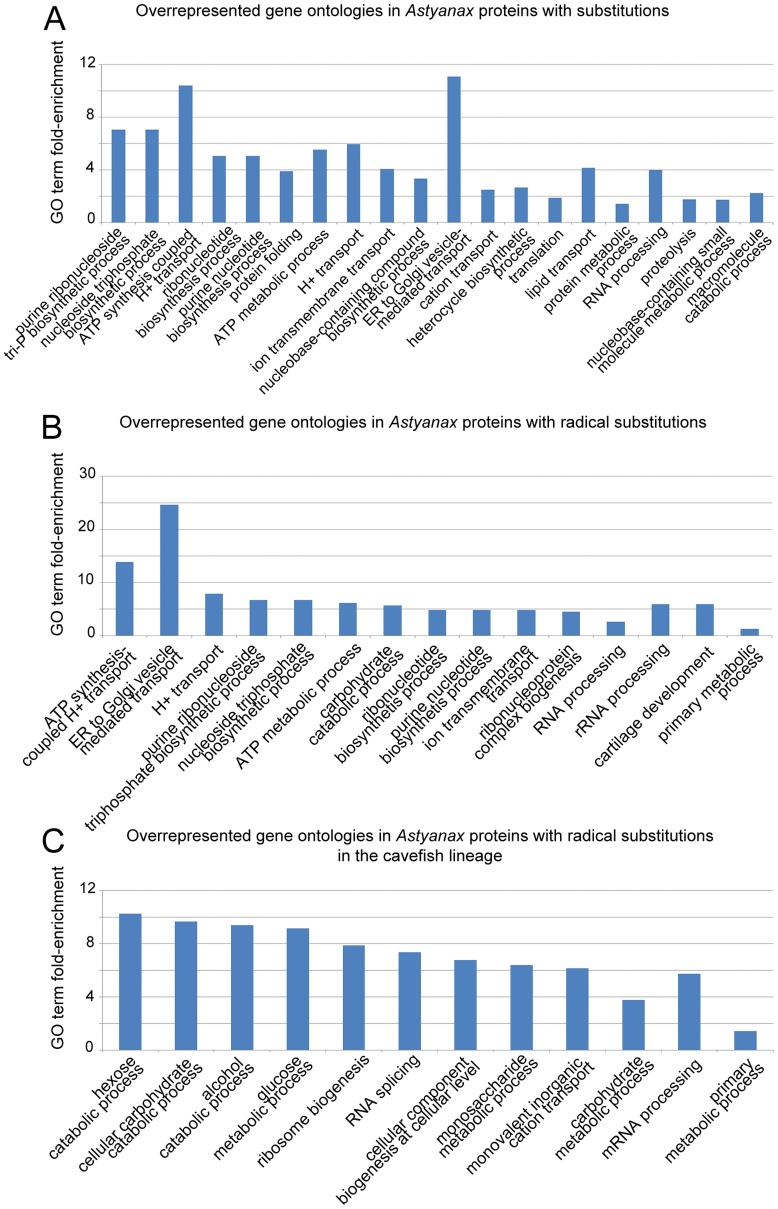
Overrepresented gene ontologies in Astyanax proteins with surface fish/cave fish substitutions (A), radical substitutions (B) or specifically with radical mutations in cavefish (C). GO terms are ordered by p-value. GO terms represented only once are not shown here.

To detect bias in the proteome evolution of surface fish and cavefish, we performed a GO term enrichment analysis on the pool of 184 genes in which the 224 substitutions were found. Surprisingly, ATP synthases seem to be over-represented among proteins with surface fish/cavefish substitutions (Fig. 4A).

Among the 224 amino acid substitutions, we found 83 radical substitutions, i.e., that correspond to amino acids with distinct physicochemical properties in the two morphs (see Methods). We performed the same analysis as above on the smaller pool of 79 genes in which the 83 radical amino acid substitutions were found, and detected the same over-representation of ATP synthases (Fig. 4B).

Within the 83 radical amino acid changes, 31 mutations had occurred in the cavefish lineage, and 22 mutations had occurred in the surface fish lineage. We found two genes for which the cavefish radical mutations are located at a highly conserved position (Fig S3): one is fkbp7 (FK506-binding protein7), a peptidyl-prolyl cis-trans isomerase which is a molecular chaperone known to bind Hsp70 in the endoplasmic reticulum [37]. Another is rpl13 (ribosomal protein L13), a component of the ribosome 60 S subunit. Fkbp7 might be an interesting candidate for phenotypic change as the deficiency in another chaperone (hsp90α) is known to play a role in the degeneration of the cavefish lens [38]. However the expression pattern of fkbp7 is unknown in fish, thus the possibility of lens function is currently speculative in *Astyanax*. Nevertheless, the high conservation of the amino acid residues that are mutated in these two proteins suggests that cavefish fkbp7 and rpl13 could be non-functional in cavefish.

Other cavefish mutations are located in conserved domains, but not at highly conserved positions: sec13, involved in protein trafficking, is mutated in a WD40 domain; capsla, the calcyphosine-like a, is mutated in a calcium-binding domain; the gametocyte specific factor 1 Gtsf1 is mutated in a zinc finger domain; and the c-Myc binding protein Mycbp is mutated in a coiled-coil domain.

We next performed a GO term enrichment analysis on proteins with radical cavefish mutations: it appears that proteins involved in carbohydrate metabolism are overrepresented (Fig. 4C). It is already known that glycogen metabolism and gluconeogenesis are different in cave and surface populations and that cavefish have a lower fasting capacity [39]. It is thus possible that the 3 proteins mutated in CF and annotated with this GO term (pgls, eno3 and chia.3) participate in this change of metabolism.

Finally, the expression patterns of the 79 transcripts with radical substitutions between surface fish and cavefish were investigated in Zfin, the zebrafish reference database [31]. Fifty-four of these transcripts had available expression patterns in Zfin. The analysis showed that 11 of the 21 ( = 52.4%) transcripts with expression annotation and carrying a mutation in cavefish are expressed in the zebrafish eye, while only 1 of the 14 ( = 7.1%) genes mutated in the surface fish lineage is expressed in this structure (Table 1). This difference is statistically significant (p = 0.00972; Fisher’s exact test). These 11 transcripts are: bcas2, fkbp3, mycbp, ndufv2, rpl13, rrp36, rrs1, eno3, capsla, sec13 and selt1a. Examples of genes mutated in cavefish, and expressed in the zebrafish visual system (retina and tectum), are shown in Fig S4 [40]. Of interest, during and after eye degeneration in Pachón, the tectum becomes largely hypomorphic [41]. The enrichment for mutations in putative eye genes in cavefish supports the hypothesis that these genes accumulate more mutations as a result of relaxed purifying selection on visual system genes in caves.

**Table 1:** Analysis of expression patterns for transcripts with radical mutations.

	number ofgenes	genes with no Zfin expression annotation	genes with Zfin expression annotation	genesexpressedin the eye	% of annotated genes	% of total
mutations in cavefish lineage	31	10	21	11	52.4%	35.5%
mutations in surface fish lineage	22	8	14	1	7.1%	4.5%
mutations not oriented	28	9	19	3	15.8%	10.7%

2 genes contain 2 mutations, one that occurred in cavefish lineage, the other in surface fish lineage: these two genes are thus counted twice in this table.

### Conclusions

We present here *de novo* Sanger sequencing of the embryonic and larval transcriptomes of *Astyanax mexicanus* surface fish and cavefish. This is the first large scale sequence resource available for the *Astyanax* research community, which will increase the usefulness of this model species in evodevo research.

We also describe genetic variations within and between the two morphs. Polymorphism in cavefish seems to be much lower than in surface fish, and we describe 940 fixed differences between surface fish and cavefish coding sequences, some of them being potentially involved in adaptation to cave life.

Among the proteins showing radical substitutions in cavefish, a third are potentially expressed in the eye, based on their expression patterns in the zebrafish *in situ* hybridization database. The accumulation of mutations in putative eye genes may be allowed because of relaxed selection for vision in the dark cave environment. These genes also represent candidates for having a role in cavefish eye degeneration. If they do have a role in the degeneration process, it would support an involvement of genetic drift as a mechanism for cavefish eye loss.

## Supporting Information

Figure S1 **Technical description of the libraries. A** Graphs showing read length frequency distribution (left) and average quality score along the reads (right) at the different stages of EST cleaning. **B** Table showing the number of EST sequences at the different stages of EST cleaning.
(TIF)

Figure S2 **Saturation curves of the libraries.** Graph showing the number of clusters of sequences as a function of the number of cDNAs sequenced.
(TIF)

Figure S3 **Local alignments of proteins mutated at a highly conserved position in cavefish.** Local alignments of fkbp7 (A) and rpl13 (B) protein orthologs in various chordate species. The position mutated in Pachón cavefish is highlighted in yellow.
(TIF)

Figure S4 **Expression patterns in zebrafish of six genes mutated in Pachón cavefish.** Zebrafish *in situ* hybridizations (ZFIN database) showing expression of ndufv2, bcas2, rrp36, rrs1, fkbp3 and sec13 in eye and tectum. Taken from Thisse et al., 2004.
(TIF)

Table S1 **List of additional transcripts (Genbank IDs) used in contig assembly (A) and detailed list of databases used for contig annotation by Blast (B).**
(DOCX)

Table S2 **Annotation statistics of the** ***Astyanax*** **contigs.**
(DOCX)
