# Supplementary figures and images for: De Novo Sequencing of Astyanax mexicanus Surface Fish and Pachón Cavefish Transcriptomes Reveals Enrichment of Mutations in Cavefish Putative Eye Genes
Source: PLoS One. 2013 Jan 9;8(1):e53553. doi: 10.1371/journal.pone.0053553 (PMC3541186; doi:10.1371/journal.pone.0053553)

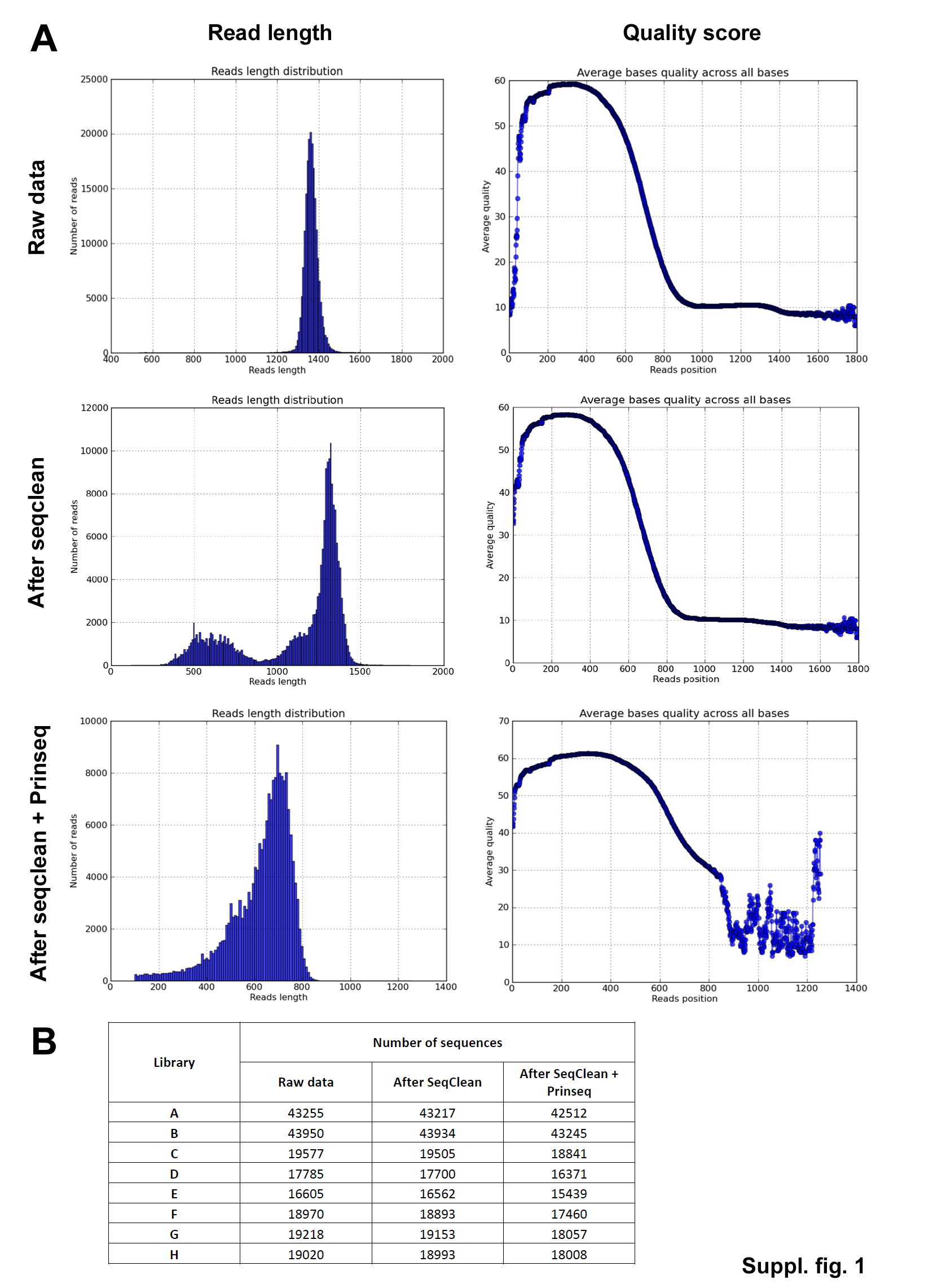

Supplement: Figure S1 — Technical description of the libraries. A Graphs showing read length frequency distribution (left) and average quality score along the reads (right) at the different stages of EST cleaning. B Table showing the number of EST sequences at the different stages of EST cleaning. (TIF) [file pone.0053553.s001.tif]

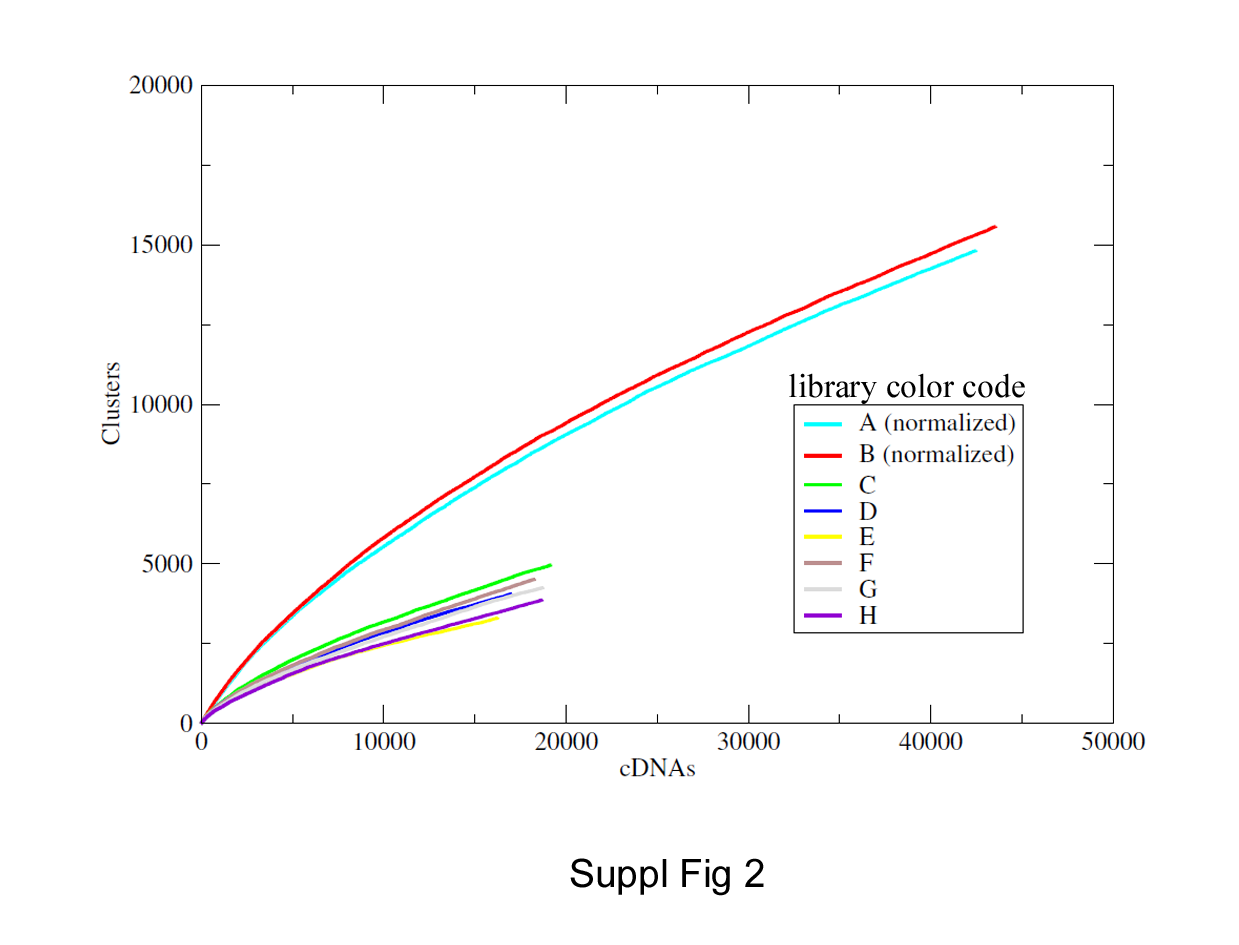

Supplement: Figure S2 — Saturation curves of the libraries. Graph showing the number of clusters of sequences as a function of the number of cDNAs sequenced. (TIF) [file pone.0053553.s002.tif]

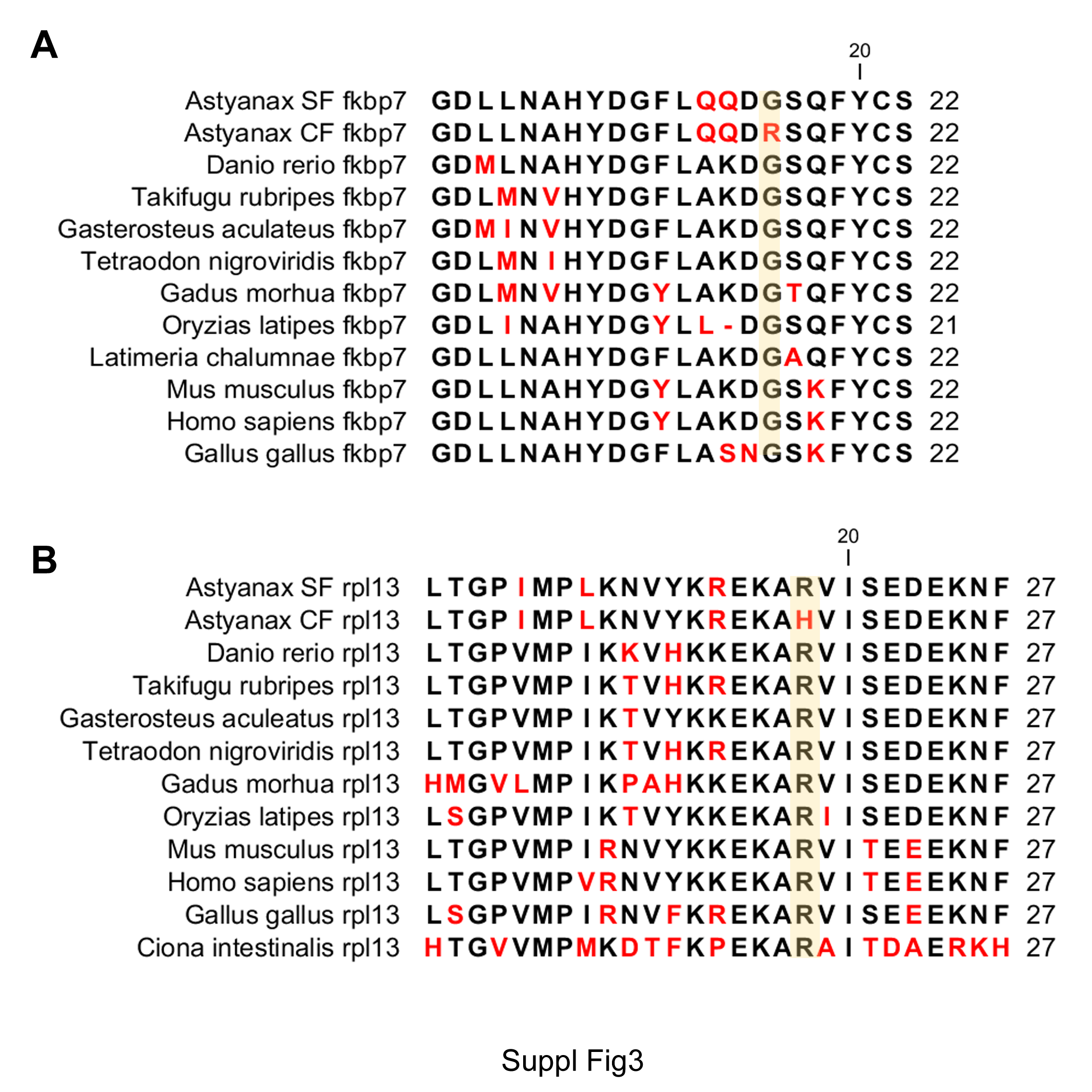

Supplement: Figure S3 — Local alignments of proteins mutated at a highly conserved position in cavefish. Local alignments of fkbp7 (A) and rpl13 (B) protein orthologs in various chordate species. The position mutated in Pachón cavefish is highlighted in yellow. (TIF) [file pone.0053553.s003.tif]

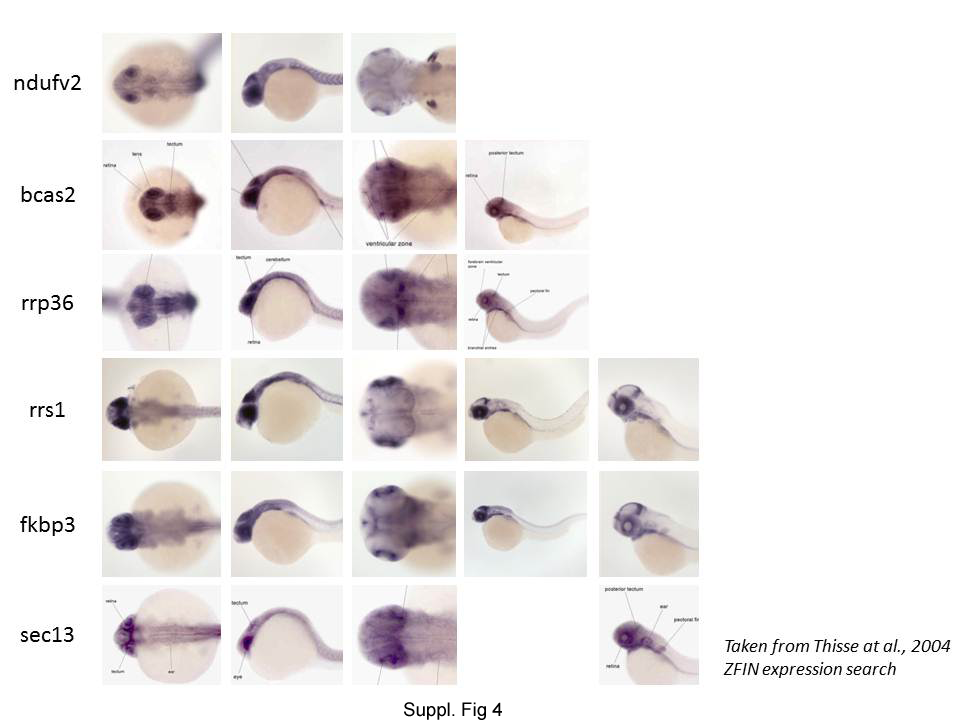

Supplement: Figure S4 — Expression patterns in zebrafish of six genes mutated in Pachón cavefish. Zebrafish in situ hybridizations (ZFIN database) showing expression of ndufv2, bcas2, rrp36, rrs1, fkbp3 and sec13 in eye and tectum. Taken from Thisse et al., 2004. (TIF) [file pone.0053553.s004.tif]
