# Supplementary material for: De Novo Sequencing of Astyanax mexicanus Surface Fish and Pachón Cavefish Transcriptomes Reveals Enrichment of Mutations in Cavefish Putative Eye Genes
Source: PLoS One. 2013 Jan 9;8(1):e53553. doi: 10.1371/journal.pone.0053553 (PMC3541186; doi:10.1371/journal.pone.0053553)
Supplement: Table S1 — List of additional transcripts (Genbank IDs) used in contig assembly (A) and detailed list of databases used for contig annotation by Blast (B). (DOCX) [file pone.0053553.s005.docx]

**Table S1: List of additional transcripts (Genbank IDs) used in contig assembly (A) and detailed list of databases used for contig annotation by Blast (B).**

A

DQ431667, DQ431668, DQ431669, DQ492288, DQ822509, DQ822510, DQ822511, DQ822512, DQ822513, DQ822514, DQ915171, DQ915172, DQ915173, EF175737, EF175738, FJ360898, FJ665983, FJ665984, FJ665985, FJ665986, HQ225730, HQ225731, HQ225732, HQ225733, HQ225734, HQ667934, Y07546, Y07547, Y07548, AF195948, AF195949, AF264702, AF264703, AY222612, AY651762, AY661431, AY661432, AY661433, AY661434, AY661435, AY661436, AY661437, AY986759

B

i\ Reference databases: UniProtKB/Swiss-Prot Release Aug-2011, UniProtKB/TrEMBL Release Aug-2011, RefSeq Protein of 31-Jul-2011, Pfam Release 25.0 of Mar-2011, RefSeq RNA of 31-Jul-2011, ii\ Species specific TIGR databases: Haplochromis_sp_red_tail_sheller HsGI 1.1, Fugu FGI 3.0, Killifish FhGI 4.0, Medaka OlGI 9.0, Salmon AsGI 6.0, Trout RtGI 8.0, ZebraFish ZGI 18.0, a_burtoni AbGI 2.1, h_chilotes HchGI 1.1, p_promelas PpGI 2.0,

iii\ UniGene species: AtlanticCod Build #11, AtlanticSalmon Build #28, BlueCatfish Build #1, ChannelCatfish Build #4, FatheadMinnow Build #12, Human Build #230, KilliFish Build #9, Medaka Build #29, NileTilapia Build #2, Pufferfish Build #9, Three-SpinedStickleback Build #6, Trout Build #32 ZebraFish Build #123

iv\ Ensembl species: Fugu Transcripts FUGU4 63,Ensembl Human Transcripts GRCh37 63, Ensembl Medaka Transcripts MEDAKA1 63, Ensembl Stickleback Transcripts BROADS1 63, Ensembl Tetraodon Transcripts TETRAODON8 63, Ensembl ZebraFish Transcripts Zv9 63.
