# Supplementary material for: De Novo Sequencing of Astyanax mexicanus Surface Fish and Pachón Cavefish Transcriptomes Reveals Enrichment of Mutations in Cavefish Putative Eye Genes
Source: PLoS One. 2013 Jan 9;8(1):e53553. doi: 10.1371/journal.pone.0053553 (PMC3541186; doi:10.1371/journal.pone.0053553)
Supplement: Table S2 — Annotation statistics of the Astyanax contigs. (DOCX) [file pone.0053553.s006.docx]

|  | Number of different annotations | Number of contigs annotated | Bit score | | | | Hit length | | | |  |  |
| --- | --- | --- | --- | --- | --- | --- | --- | --- | --- | --- | --- | --- |
|  |  |  | Mean | Median | Minimum | Maximum | Mean | Median | Minimum | Maximum |  |  |
| Swissprot | 10858 | 20956 | 509 | 431 | 53 | 3994 | 433 | 395 | 47 | 2573 | 23423 contigs (53.1%) with proteic annotation | 40288 contigs (91.3%) with an annotation |
| TrEMBL | 13587 | 23134 | 564 | 487 | 87 | 4056 | 435 | 395 | 47 | 2573 |  |  |
| RefSeq Prot | 12228 | 23025 | 559 | 484 | 51 | 4056 | 435 | 398 | 47 | 2573 |  |  |
| RefSeq RNA | 20560 | 20890 | 119 | 86 | 28 | 1235 | 301 | 239 | 28 | 2588 | 39855 contigs (90.3%) with nucleotidic annotation |  |
| Tigr | 23941 | 35733 | 81 | 47 | 18 | 782 | 196 | 106 | 17 | 1952 |  |  |
| Unigene | 23704 | 37767 | 85 | 48 | 18 | 969 | 195 | 105 | 17 | 2487 |  |  |

**Table S2 : Annotation statistics of the *Astyanax* contigs**
